# Editorial Comment to Bladder stone formation around polyethylene glycol after use of SpaceOAR hydrogel

**DOI:** 10.1002/iju5.12634

**Published:** 2023-09-01

**Authors:** Masashi Morita

**Affiliations:** ^1^ Department of Urology Showa University Koto Toyosu Hospital Tokyo Japan

SpaceOAR is currently the only hydrogel spacer available in Japan and has been actively used in conjunction with radiation therapy for prostate cancer since its introduction in 2018.

A multicenter randomized controlled trial involving its use with IMRT showed a significant reduction in the frequency of Grade 2 or higher late rectal toxicities.^1^ The reports also included descriptions of the ease of the procedure, despite their initial experience, with 98.7% of operators rating it as “very easy” or “easy.” However, while the procedure is perceived as easy, the number of reported procedure‐related adverse events are increasing each year. In some cases, the radiation therapy itself could not be performed due to high‐grade procedure‐related adverse events. High‐grade adverse events are frequently attributed to the gel misplacement within the rectal wall caused by intraoperative manipulation, leading to rectal ulcers or fistulas. The problem with such ectopic gel placement is that, although it is probably caused by some operation during the procedure, most cases end the procedure without noticing any problems.

In this study, the authors report a rare case of gel misplacement into the bladder and the subsequent formation of a bladder stone within a short period.^2^ Unfortunately, the details of the procedure are unknown, and the specific cause of the ectopic placement into the bladder remains unclear. However, if the cause is the indwelled gels in the seminal vesicles, as the authors speculated, it is highly possible that puncture of the seminal vesicles occurred during the puncture process, and it should be noted that such calculus formation may occur afterward. Therefore, it is important to carefully perform each procedure process, and it may also be important to objectively confirm the indwelling state by MRI early after the procedure. Early detection of ectopic placement may allow action to be taken to avoid high‐grade adverse events.

## Author contributions

Masashi Morita: Writing – original draft; writing – review and editing.

## Conflict of interest

The author declares no conflict of interest.
